# 491. Risk Factors for Severe Pediatric COVID-19: A Systematic Review

**DOI:** 10.1093/ofid/ofad500.560

**Published:** 2023-11-27

**Authors:** Carlos R Oliveira, Zachary I Willis, Gabriela Maron, Paul K Sue, Brenda I Anosike, Laura Bio, Prachi Singh, Scott H James, Christine M Miller, Mari M Nakamura, Joshua Wolf

**Affiliations:** Yale University, New Haven, Connecticut; University of North Carolina at Chapel Hill School of Medicine, Chapel Hill, North Carolina; St. Jude Children's Research Hospital, Memphis, TN; UT Southwestern Medical Center, Dallas, Texas; Children's Hospital at Montefiore, The Bronx, New York; Lucile Packard Children's Hospital Stanford, Palo Alto, CA; UCSF Benioff Children's Hospital Oakland, Oakland, California; University of Alabama at Birmingham, Birmingham, Alabama; Yale University School of Medicine, New Haven, Connecticut; Boston Children's Hospital/Harvard Medical School, Boston, Massachusetts; St. Jude Children's Research Hospital, Memphis, TN

## Abstract

**Background:**

Optimal management of COVID-19 in children requires risk stratification based on comorbidities and demographic factors that can predispose to severe disease. The Pediatric Infectious Diseases Society (PIDS) Pediatric COVID-19 Therapies Task Force, comprised of pediatric infectious diseases physicians, intensivists, and pharmacists from 29 US hospitals, develops clinical guidance for pediatric COVID-19 management. In support of these efforts, a systematic review of peer-reviewed literature was conducted to synthesize the evidence for risk factors for severe pediatric COVID-19.

**Methods:**

Medline, EMBASE, and CDC databases were searched to identify all relevant publications before July 1, 2022. Titles and abstracts were reviewed to identify studies that assessed for potential predictors of severe COVID-19 disease in children < 21 years. Severe disease was defined by intensive care unit admission, invasive mechanical ventilation, multiorgan dysfunction, or death. A team of reviewers appraised eligible studies, extracted relevant data, and assessed the quality of evidence. Comorbidities and demographic factors were classified as **definite**, **probable**, or **unlikely** risk factors based on the certainty of association with severe COVID-19.

**Results:**

Sixteen potential risk factors were evaluated based on evidence from 50 studies: 13 reviews/meta-analyses, 23 multi-center, and 14 single-center studies (Figure 1). Severe immunocompromise, obesity, diabetes, prematurity, and neurologic, cardiovascular, and chronic lung disease were classified as definite risk factors. Evidence was less consistent in support of sickle cell disease, mild/moderate immunocompromise, neurodevelopmental, and chronic liver disorders as risk factors. Most studies found asthma, sex, mental health, chronic kidney disease, and inflammatory bowel disease to be unlikely risk factors. Many studies demonstrated that the magnitude of risk for comorbidities was modified by prior immunization, age, and medical complexity (i.e., multiple or poorly controlled comorbidities) (Figure 2).

**Figure 1.**
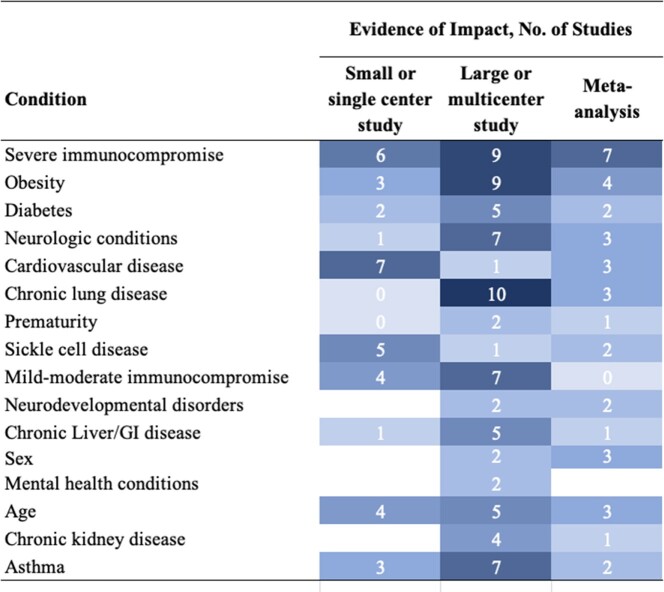
Evidence Review: Comorbidities and Severe COVID-19 in Children

Obesity - BMI ≥95th percentile for age and sex per CDC growth curves. Severe immunocompromise - Any of the following: Receipt in the 3 months before COVID-19 diagnosis of chemotherapy for a solid tumor or hematologic malignancy, high-dose corticosteroids (e.g., prednisone >20mg/day for ≥14 days), or other systemic B- or T-cell-depleting immunosuppressive agents; hematopoietic stem cell transplant, CAR T cell therapy, or solid organ transplant within 100 days of COVID-19 diagnosis; human immunodeficiency virus infection and CD4 count <200; combined primary immunodeficiency disorders. Moderate immunocompromise - Routine receipt of non-lymphocyte-depleting immunosuppressive or immunomodulatory medications or prednisone <20 mg/day for inflammatory/immune-mediated disease.

**Figure 2.**
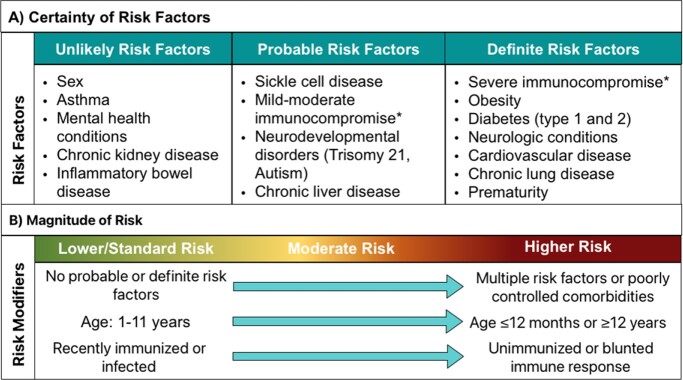
Risk Factors and Risk Modifiers for Severe Pediatric COVID-19 *Children who are less likely to gain protection from previous infections or vaccines. Definite risk factor - Evidence for increased risk for severe COVID-19, supported by large multicenter studies, meta-analyses, or systematic reviews. Probable risk factor - Evidence for increased risk, supported by small or single-center studies; this category also includes factors inconsistently associated with severe COVID-19, suggesting substantial uncertainty. Unlikely risk factor - Evidence against increased risk for severe COVID-19, supported by large multicenter studies, meta-analyses, or systematic reviews.

**Conclusion:**

This study highlights key comorbidities and effect modifiers associated with severe COVID-19 in children. These findings can be used to facilitate risk stratification and inform management decisions.

**Disclosures:**

**Zachary I. Willis, MD, MPH**, Merck Sharp & Dohme Corp: Grant/Research Support|Pfizer Inc: Grant/Research Support **Gabriela Maron, MD**, Astellas Inc: Grant/Research Support|SymBio Pharma: Grant/Research Support **Paul K. Sue, MDCM**, Allovir, Inc: Participant in Industry Sponsored Trial|Gilead Sciences, Inc: Participant in Industry Sponsored Trial|Merck & Co.: Participant in Industry Sponsored Trial **Scott H. James, MD**, Bayer: Advisor/Consultant|Evrys: Grant/Research Support|Gilead: Grant/Research Support **Mari M. Nakamura, MD, MPH**, Gilead Sciences, Inc.: Grant/Research Support **Joshua Wolf, MBBS, PhD**, Karius Inc.: Grant/Research Support|Merck Inc.: Participation in industry-sponsored research

